# Impact of enzyme replacement therapy and migalastat on disease progression in females with fabry disease

**DOI:** 10.1186/s13023-025-03600-y

**Published:** 2025-02-20

**Authors:** Malte Lenders, Albina Nowak, Markus Cybulla, Jessica Kaufeld, Anja Friederike Köhn, Nicole Maria Muschol, Christine Kurschat, Eva Brand

**Affiliations:** 1 Department of Internal Medicine D, and Interdisciplinary Fabry Center (IFAZ), University Hospital Muenster, Muenster, Germany; 2 Department of Endocrinology and Clinical Nutrition, University Hospital Zuerich and University of Zuerich, Zuerich, Switzerland; 3 Department of Nephrology and Rheumatology, Nephrologicum-MGL MVZ, Muellheim, Germany; 4 Department of Nephrology and Hypertension, Hannover Medical School, Hannover, Germany; 5 International Center for Lysosomal disorders (ICLD), Department of Pediatrics, University Medical Center Hamburg-Eppendorf, Hamburg, Germany; 6 Department II of Internal Medicine, Center for Molecular Medicine Cologne and Center for Rare Diseases, University of Cologne, Cologne, Germany

**Keywords:** Females, Fabry disease, Enzyme replacement therapy, Migalastat, Disease progression

## Abstract

**Aim:**

The aim of our multicenter study was to investigate the safety and efficacy of enzyme replacement therapy (ERT) and chaperone therapy on the disease progression in female Fabry disease (FD) patients and to compare the individual treatment regimens.

**Methods:**

Data from 3 consecutive visits of 102 female FD patients from 6 Fabry centers were retrospectively analyzed. According to their FD-specific treatment, patients were separated in 5 groups: Newly agalsidase-beta- [*n* = 18], agalsidase-alfa- [*n* = 29] and migalastat-[*n* = 14] treated patients, and long-term agalsidase-beta- [*n* = 7] and agalsidase-alfa-[*n* = 34] treated patients. Clinical presentation and laboratory data, including plasma lyso-Gb_3_ levels were assessed.

**Results:**

Treatment with agalsidase-beta, agalsidase-alfa, and migalastat was safe and severe adverse events were rare. Newly and long-term-treated patients presented a stable disease course over time. None of the patients required hospitalization due to cardiac events. Overall septum thickness remained stable in all groups (*p* > 0.05). Estimated glomerular filtration rate (eGFR) only slightly decreased in patients treated with agalsidase-alfa [newly- and long-term-treated: -1.5 ± 3.2 and − 1.3 ± 3.9 ml/min/1.73 m²/year; *p* = 0.0056 and *p* = 0.0187, respectively] but the decrease was in the range of natural eGFR decline. eGFRs in agalsidase-beta and migalastat-treated patients were stable. No clinically relevant differences concerning treatment efficacy between agalasidase-beta, agalsidase-alfa, and migalastat were detected.

**Conclusion:**

We conclude that treatment of females with agalsidase-beta, agalsidase-alfa, and migalastat was safe. Independent of the chosen treatment regimen, nearly all patients presented with a stable disease course over time. In our cohort, a comparison of therapy efficacies showed no relevant clinical differences between the groups.

**Supplementary Information:**

The online version contains supplementary material available at 10.1186/s13023-025-03600-y.

## Introduction

Fabry disease (FD; OMIM #301500) is an X-linked inherited lysosomal storage disease due to deficient α-galactosidase A activity (AGAL/GLA) based on pathogenic mutations within the *GLA* gene. The resulting FD-specific symptoms and manifestations originate from systemic cellular lysosomal accumulation of mainly globotriaoslyceramide (Gb_3_) [Zarate, Hopkin 2008]. The progressive lysosomal Gb_3_ accumulation results in increased risks of an early onset of stroke, life-threatening arrhythmia and myocardial infarction, and/or cardiac and renal failure, eventually leading to a reduced life expectancy in affected males and females [1].

Female FD patients are heterozygous for the pathogenic *GLA* mutation and show X-chromosomal inactivation, thus, they present with a broader clinical phenotypic variability compared to affected males [2]. In this respect, female patients can be asymptomatic or show only mild symptoms. In general, the symptoms and manifestations appear later in females than in affected males.

Manifestations in affected females are heterogeneous, and FD-specific therapy (in contrast to prophylactic therapy in classical males) is only given once an organ manifestation (renal, cardiac or neurological) has been detected [3]. Therefore, longitudinal observational data focusing on females under FD-specific therapy are required to analyze the effect of treatment in these patients [2]. Of note, a recent study of kidney tissue demonstrated that Gb_3_ accumulation in affected podocytes progresses with age in females, too, and it is associated with podocyte loss and proteinuria [4]. Importantly, the observed Gb_3_ accumulation in affected females was comparable to that in males [4], confirming the lack of cross-correction between affected and unaffected cells in females [5, 6]. Thus, the pathological processes in females and males appear comparable, but many affected females with FD are not treated accordingly yet [7, 8].

Furthermore, with regard to the currently available treatment options (agalsidase-alfa [0.2 mg/kg body weight, intravenously every other week [e.o.w.]], agalsidase-beta [1.0 mg/kg body weight, intravenously e.o.w.], pegunigalsidase-alfa [1.0 mg/kg body weight, intravenously e.o.w.] and migalastat [123 mg, orally every other day]), it is important to compare the effectiveness of treatment in terms of disease progression and the varying costs to the healthcare system. In this respect, enzyme replacement therapy (ERT) can be used to treat all patients with different mutations, while migalastat is only approved for patients with amenable missense mutations. Since patients with amenable missense mutations usually have residual enzymatic activity, this often leads to a milder disease burden in the affected patients [3]. This should be taken into account when interpreting the comparison of different treatment groups.

In this study, we evaluated a cohort of 102 genetically confirmed female FD patients from six Fabry centers (five German centers, one Swiss center) with well-characterized clinical phenotypes at three time points to assess the impact of agalsidase-alfa, agalsidase-beta and migalastat in newly- as well as long-term-treated patients. Furthermore, we also analyzed and compared the effectiveness of the individual treatment regimens.

## Methods

**Fig. 1:**
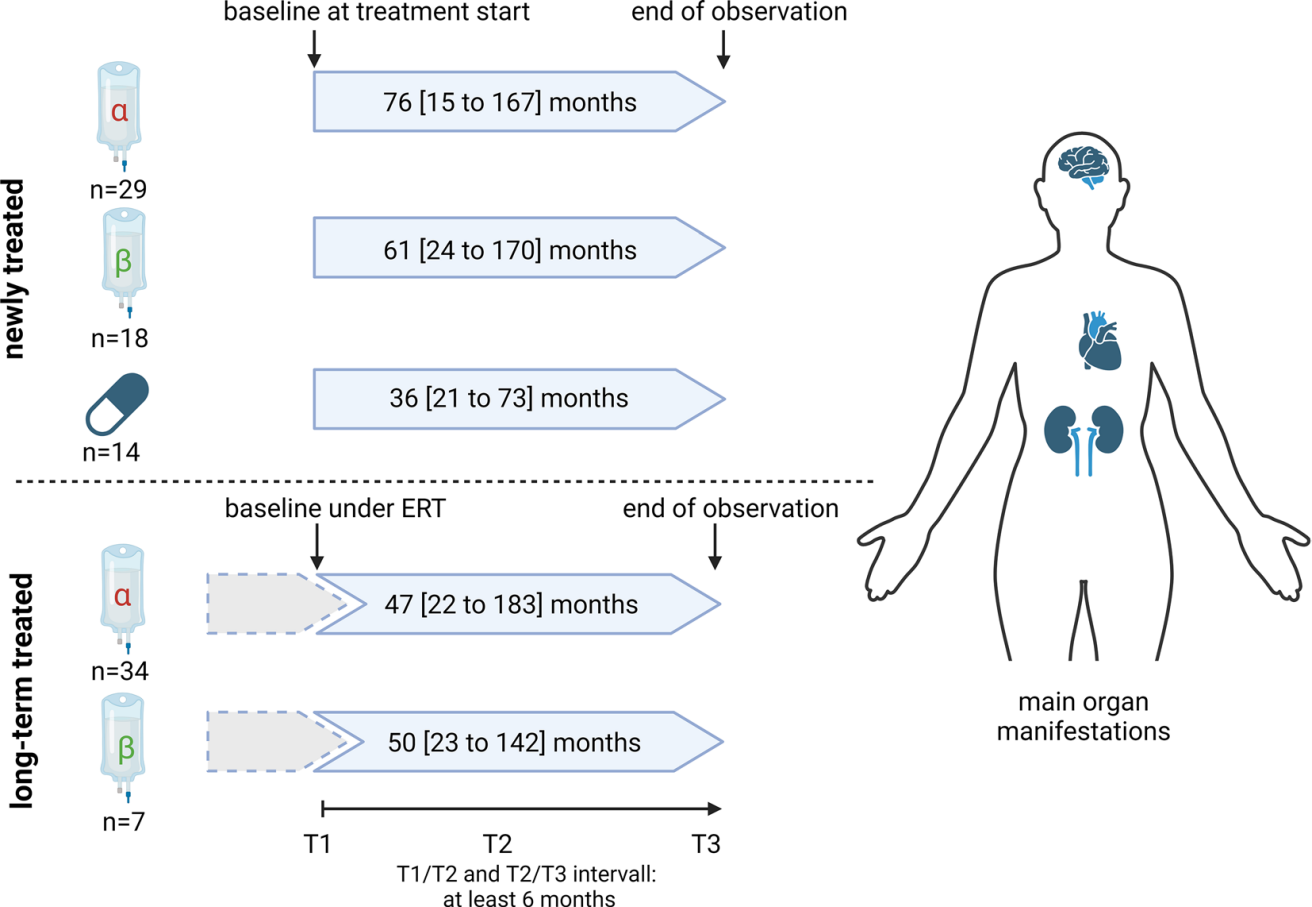
Overview of the study design. In total, 61 female patients with genetically confirmed Fabry disease (FD) were consecutively recruited and newly treated with either agalsidase-alfa (α) [*n* = 29], agalsidase-beta (β) [*n* = 18], or migalastat [*n* = 14]. In addition, 41 females already long-term treated with enzyme replacement therapy (ERT) (agalsidase-alfa, *n* = 34; agalsidase-beta, *n* = 7) were recruited and observed over time. Participating centers: Muenster (n=39), Cologne (n=20), Zuerich (n=26), Muellheim (n=9), Hannover (n=4), Hamburg (n=4).

A total of 102 genetically confirmed adult female FD patients were consecutively recruited at the German Fabry centers of the University Hospitals in Muenster (n=39), Cologne (n=20), Hamburg (n=4) and Hannover (n=4), as well as in the Fabry Center in Muellheim (n=9) and Zuerich (n=26) in Switzerland (Fig. 1). All investigations were performed after approval by the respective ethics committees of the participating centers (leading committee: Medical Association of Westfalian-Lippe and the Ethical Committee of the Medical Faculty of the University of Muenster; project number: 2016-401-f-S; 2011-347-f) and written informed consent for molecular analysis and publication was obtained from all patients, where appropriate.

Patients were retrospectively analyzed at three time points (T1 [baseline], T2 [in between], T3 [end of observation]). Inclusion criteria for this study were as follows: (i) female patient ≥ 18 years (at time point T3) and a genetically confirmed disease-causing *GLA* mutation, (ii) naïve to any FD-specific therapy (ERT or migalastat) or on a stable dose of agalsidase-alfa or agalsidase-beta for at least 6 months at baseline, (iii) an interval of at least 6 months between 3 consecutive visits (T1 to T2 and T2 to T3), (iv) patient currently not in any clinical trial.

The group of newly-treated females includes patients being therapy-naïve to any FD-specific treatment at their first visit (baseline/T1) and started an appropriate therapy (agalsidase-alfa, agalsidase-beta and migalastat) after T1. The group of long-term ERT-treated females includes patients already being treated with either agalsidase-alfa or -beta at study start. In these patients, baseline/T1 was determined as the time point under ongoing therapy with comparable periods between T1 and T3 compared to the newly-treated females described above. Visits at T2 (in between) and T3 (end of observation) were during the treatment (Fig. 1). Patients with mutations of unknown significance including p.S126G, p.A143T, and p.D313Y were not included. A detailed overview of all detected *GLA* mutations is provided within Supplemental Table S1. Nonsense mutations were defined as single nucleotide exchanges, resulting in stop codons (termination), deletions or insertions of nucleotides resulting in a frame shift or large deletions within the protein, or splice site mutations, resulting in altered splice products of mRNA.

A comprehensive diagnostic work-up as listed in Supplemental Table S2 was performed in all patients at each visit, including medical history and cardiac, renal, and neurologic evaluation. The data documentation followed the clinical practice of the interdisciplinary Fabry expert centers. The detailed clinical work-up of patients was reported previously [7]. Due to the COVID19 pandemic and the retrospective approach, the optional MRIs to assess data for cardiac and cerebral manifestations were limited and, thus, not evaluated.

Gastrointestinal (GI) symptoms include abdominal pain, tenesmus, or cramps more than once a week. Diarrhea was defined as ≥ 1 day/ month with three loose bowels or > 250 g of stool weight per day. Fatigue was defined by the Fukuda criteria [9]. Cardiac assessment included echocardiography with left ventricular hypertrophy (LVH) defined as an interventricular septum thickness in diastole (IVSd) ≥ 11.5 mm [10]. Renal function was quantified by eGFR using the Chronic Kidney Disease - Epidemiology Collaboration equation (CKD-EPI) based on serum creatinine [11] and the albumin-to-creatinine ratio (ACR) from morning spot urine. Renal impairment was defined as an eGFR < 90 ml/min/1.73 m^2^ according to European FD guidelines [12] and albuminuria as ACR > 30 mg albumin per g creatinine. Patients underwent neurological examination and a clinical interview focusing on a history of cerebral stroke or transient ischemic attack (TIA). Additionally, the presence of FD-related pain was investigated [13]. Disease severity was assessed using the Disease Severity Scoring System (DS3) [10] and the Mainz Severity Score Index (MSSI) [14]. Additional concomitant medication was assessed for every visit. Renin-angiotensin-aldosterone system (RAAS) blockers include the prescription of angiotensin-converting enzyme blockers, angiotensin receptor blockers, renin blockers, as well as aldosterone receptor antagonists. Diuretics include the prescription of high ceiling/loop diuretics, thiazides, carbonic anhydrase inhibitors, and potassium-sparing diuretics. Analgesic drugs include the prescription of opioids, anticonvulsants, selective serotonin reuptake inhibitors, and non-steroidal anti-inflammatory drugs.

Plasma lyso-Gb_3_ levels (normal ≤ 1.8 ng/ml) were measured at Centogene (Rostock, Germany) or Archimed Life Science GmbH (Vienna, Austria).

## Statistical analysis

If not stated otherwise, continuous variables are expressed as median with range or minimum to maximum, since most data were unequally distributed. Categorical data are expressed as numbers and relative frequencies in percent. A quality control of assessed data is provided in the supplements (Supplemental Table S3) showing an overall data completeness of 94.0%. To deal with missing data, analyses were performed with an as-is state for every parameter with individual patient numbers provided within tables and figures. Differences in a group between the three visits (T1, T2, and T3) were analyzed with a Friedman test, and differences between groups were analyzed with the Kruskal-Wallis test for continuous data. A Chi-square test was used for categorical data. Fisher’s exact test was used to calculate the relative risk (RR) for concomitant medications between T1 and T3. Mean changes for IVSd, eGFR, lyso-Gb_3_, DS3 and MSSI were individually calculated based on the three consecutive values from T1 to T3 and significant changes were analyzed using the Wilcoxon Signed Rank test. Statistical significance was considered at a 2-sided *p* < 0.05. SAS version 9.4 (SAS Institute Inc., Cary, North Carolina, USA) and GraphPad PRISM V8.4 software (GraphPad Software Inc., La Jolla, CA, USA) were used for statistical analyses and data visualization. Cartoons were created with BioRender.com.

## Results

### Clinical characterization of female patients with Fabry disease

To assess the end-organ damage and impact of ERT (including treatment with agalsidase-alfa [0.2 mg/ kg i.v. body weight every other week], agalsidase-beta [1.0 mg/ kg i.v. body weight every other week]) or chaperone therapy (migalastat, 123 mg orally every other day), 102 female patients with genetically confirmed FD were recruited at 6 FD centers (Fig. 1). Patients’ data were assessed from 3 consecutive visits T1 (baseline), T2 (in between), and T3 (end of observation). According to their FD-specific treatment, patients were separated in 5 groups: (i) newly agalsidase-beta-treated patients [*n* = 18], (ii) newly agalsidase-alfa-treated patients [*n* = 29], (iii) newly migalastat-treated patients [*n* = 14] (patients within the groups i) to iii) were naïve to any FD-specific treatment at their first visit at baseline), (iv) long-term (ERT before baseline and during follow-up) agalsidase-beta -treated patients [*n* = 7] or (v) long-term agalsidase-alfa-treated patients [*n* = 34] (Fig. 1).

Baseline characteristics of the newly treated patients are presented in Table 1. All three groups showed comparable symptoms and manifestations, suggesting a similar disease burden at baseline. However, newly ERT-treated patients (including agalsidase-alfa and -beta) presented with higher plasma lyso-Gb_3_ at baseline compared to those receiving migalastat (*p* = 0.0046; Table 1). Baseline characteristics of the long-term ERT-treated patients revealed no significant differences between agalsidase-beta- and agalsidase-alfa-treated patients at baseline, too (Table 2).

**Table 1:** Treatment-naïve baseline characteristics of the newly treated female patients

	*n*	Agalsidase-beta	*n*	Agalsidase-alfa	*n*	Migalastat	*p* value
age, years	18	49.0 [25.0 to 71.0]	29	49.0 [25.0 to 73.0]	14	53.0 [23.0 to 72.0]	0.5100
BMI, kg/m²	18	26.6 [20.8 to 35.5]	28	24.3 [18.8 to 34.0]	14	23.8 [19.0 to 50.8]	0.2349
SBP, mmHg	18	130 [110 to 150]	27	140 [99 to 199]	14	125 [93 to 160]	0.1311
DBP, mmHg	18	82 [65 to 90]	27	80 [52 to 106]	14	75 [67 to 95]	0.2229
nonsense mutations, n	18	9 (50.0)^#^	29	21 (72.4)^#^	14	0 (0.0)	**0.0001**
hypertension, n	18	4 (22.2)	27	10 (37.0)	14	2 (14.3)	0.2555
lyso-Gb_3_, ng/ml	12	10.1 [4.8 to 19.3]^#^	19	9.5 [3.4 to 17.8]^#^	14	5.1 [0.9 to 11.1]	**0.0046**
lyso-Gb_3_ > reference, n	12	12 (100.0)	19	19 (100.0)	14	12 (85.7)	0.0985
edema, n	18	2 (11.1)	30	1 (3.3)	13	1 (7.7)	0.5640
GI symptoms, n	15	6 (40.0)	27	5 (18.5)	11	5 (45.4)	0.1615
angiokeratoma, n	18	5 (27.8)	28	14 (50.0)	14	5 (35.7)	0.3021
fatigue, n	16	4 (25.0)	25	3 (12.0)	8	2 (25.0)	0.5376
FD-related pain, n	18	12 (66.7)	29	18 (62.1)	14	9 (64.3)	0.9499
TIA/stroke, n	18	3 (16.7)	29	3 (10.3)	14	3 (21.4)	0.6076
DS3 total score	18	10 [1 to 26]	24	9 [3 to 20]	14	10 [0 to 34]	0.6428
MSSI total score	18	17 [4 to 29]	27	10 [2 to 25]^#^	14	18 [7 to 31]	**0.0238**
IVSd, mm	18	13.4 [8.0 to 22.0]	23	11.0 [8.0 to 26.0]	12	13.5 [5.0 to 24.0]	0.8366
LVH, n	18	11 (61.1)	23	8 (34.8)	12	7 (58.3)	0.1887
pacemaker/ICD, n	18	1 (5.5)	29	1 (3.4)	14	2 (14.3)	0.3962
serum creatinine, mg/dl	18	0.78 [0.46 to 1.84]	29	0.70 [0.46 to 1.10]	14	0.75 [0.50 to 1.07]	0.4132
eGFR, ml/min/1.73 m²	18	92 [33 to 135]	29	99 [65 to 131]	14	89 [53 to 128]	0.6045
CKD G1		10 (55.5)		19 (65.5)		6 (42.8)	0.3648
CKD G2		7 (38.9)		10 (34.5)		7 (50.0)	0.6204
CKD G3		1 (5.5)		0 (0.0)		1 (7.1)	0.3797
ACR, mg/g protein	14	59 [6 to 2436]	22	39 [0 to 279]	11	25 [0 to 992]	0.1727
albuminuria, n	14	11 (78.6)	22	13 (59.1)	11	5 (45.4)	0.2256
dialysis, n	18	0 (0.0)	29	0 (0.0)	14	0 (0.0)	0.9999
KTX, n	18	1 (5.5)	29	0 (0.0)	14	0 (0.0)	0.2969
RAAS blocker, n	16	3 (18.7)	27	13 (48.1)	14	6 (42.8)	0.1491
diuretics, n	16	1 (6.2)	28	4 (14.3)	14	2 (14.3)	0.7029
analgesics, n	15	3 (20.0)	26	1 (3.8)	14	3 (21.4)	0.1725

Albuminuria was defined as an albumin/creatinine ratio (ACR) > 30 mg albumin per gram of creatinine from spot urine. Nonsense mutations include the presence of nonsense mutations, insertions/deletions and intronic mutations. BMI: body mass index, CKD: chronic kidney disease, DBP: diastolic blood pressure, DS3: disease severity scoring system, eGFR: estimated glomerular filtration rate, ERT: enzyme replacement therapy (includes treatment with agalsidase-alfa or -beta). FD: Fabry disease, GI: gastrointestinal symptoms including the presence of diarrhea and/or abdominal pain, ICD: implantable cardioverter device, IVSd: interventricular septum thickness in diastole, KTX: kidney transplantation, lyso-Gb_3_: globotriaosylsphingosine with an upper limit of normal of 1.9 ng/ml, LVH: left ventricular hypertrophy, defined as IVSd > 11.5 mm, MSSI: Mainz severity score index, RAAS: renin-angiotensin-aldosterone system, SBP: systolic blood pressure, TIA: transient ischemic attack. ^#^*p* < 0.05 versus migalastat-treated

**Table 2:** Baseline characteristics under therapy at study start of the long-term ERT-treated female patients

	*n*	Agalsidase-beta	*n*	Agalsidase-alfa	*p*-value
age, years	7	48 [16 to 63]	34	47 [17 to 75]	0.7154
pretreated since, months	7	72 [41 to 89]	34	95 [7 to 189]	0.1935
BMI, kg/m²	7	21.9 [18.3 to 33.5]	33	24.9 [18.6 to 38.3]	0.5333
SBP, mmHg	6	117 [109 to 150]	32	118 [100 to 152]	0.7771
DBP, mmHg	6	74 [58 to 100]	32	69 [56 to 93]	0.6456
nonsense mutations, n	7	4 (57.1)	34	17 (50.0)	0.9999
hypertension, n	6	2 (33.3)	32	4 (12.5)	0.2341
lyso-Gb_3_, ng/ml	5	8.1 [2.5 to 11.3]	25	8.2 [2.5 to 14.5]	0.9231
lyso-Gb_3_ > reference, n	5	5 (100.0)	25	25 (100.0)	0.9999
edema, n	7	2 (28.6)	34	6 (17.6)	0.6059
GI symptoms, n	7	0 (0.0)	34	4 (11.8)	0.9999
angiokeratoma, n	7	4 (57.1)	34	18 (52.9)	0.9999
FD-related pain, n	7	4 (57.1)	34	25 (73.5)	0.3978
TIA/stroke, n	7	1 (14.3)	34	8 (23.5)	0.9999
DS3 total score	7	9 [2 to 26]	32	9 [1 to 26]	0.7947
MSSI total score	7	17 [4 to 27]	33	17 [3 to 37]	0.6947
IVSd, mm	5	11.0 [7.0 to 12.0]	29	10.0 [7.0 to 21.0]	0.7349
LVH, n	5	2 (40.0)	29	12 (41.4)	0.9999
pacemaker/ICD, n	7	0 (0.0)	34	0 (0.0)	0.9999
serum creatinine, mg/dl	5	0.64 [0.60 to 0.68]	33	0.73 [0.49 to 1.24]	**0.0287**
eGFR, ml/min/1.73 m²	5	97 [95 to 135]	32	97 [43 to 142]	0.3153
CKD G1		5 (100.0)		22 (68.7)	0.2954
CKD G2		0 (0.0)		9 (28.1)	0.3067
CKD G3		0 (0.0)		1 (3.1)	0.9999
ACR, mg/g protein	6	38 [0 to 853]	31	27 [0 to 1443]	0.8803
albuminuria, n	6	3 (50.0)	31	14 (45.2)	0.9999
dialysis, n	7	0 (0.0)	34	0 (0.0)	0.9999
KTX, n	7	0 (0.0)	34	0 (0.0)	0.9999
RAAS blocker, n	6	3 (50.0)	34	14 (41.2)	0.9999
diuretics, n	6	1 (16.7)	34	5 (14.7)	0.9999
analgesics, n	6	1 (16.7)	32	5 (15.6)	0.9999

Albuminuria was defined as an albumin/creatinine ratio (ACR) > 30 mg albumin per gram of creatinine from spot urine. Nonsense mutations include the presence of nonsense mutations, insertions/deletions and intronic mutations. BMI: body mass index, CKD: chronic kidney disease, DBP: diastolic blood pressure, DS3: disease severity scoring system, eGFR: estimated glomerular filtration rate, ERT: enzyme replacement therapy (includes treatment with agalsidase-alfa or -beta). FD: Fabry disease, GI: gastrointestinal symptoms including the presence of diarrhea and/or abdominal pain, ICD: implantable cardioverter device, IVSd: interventricular septum thickness in diastole, KTX: kidney transplantation, lyso-Gb_3_: globotriaosylsphingosine with an upper limit of normal of 1.8 ng/ml, LVH: left ventricular hypertrophy, defined as IVSd > 11.5 mm, MSSI: Mainz severity score index, RAAS: renin-angiotensin-aldosterone system, SBP: systolic blood pressure, TIA: transient ischemic attack

### Safety and therapy efficacy of enzyme replacement and chaperone therapy

The duration of observation periods did not significantly differ between the groups (newly treated: agalsidase-alfa: 76 [15 to 167] months, agalsidase-beta: 61 [24 to 170] months, migalastat: 36 [21 to 73] months; *p* = 0.0689; long-term-treated: agalsidase-alfa: 47 [22 to 183] months, agalsidase-beta: 50 [23 to 142] months; *p* = 0.7281). However, the observation period was shortest for those patients receiving migalastat (Fig. 1).

First, we assessed the safety of all FD-specific therapies. None of the patients suffered from severe adverse events (including anaphylactic shock, rush, shivering, hypotonic crises) during the observational period. In newly agalsidase-beta and -alfa-treated females, only four patients required pre-medication with paracetamol, cortisone, and histamine type-2 receptor antagonists (H2 blockers) each, due to an allergic reaction to the infused enzyme. The pre-medication could be tapered within ongoing treatment and was completely suspended after T2 in three females (one of which was agalsidase-beta-treated).

Next, we analyzed frequencies of cerebrovascular events including TIAs and strokes between baseline and end of observation. During baseline and end of observation, two events occurred in newly agalsidase-beta-treated patients, three events occurred in newly agalsidase-alfa-treated patients, and one event occurred in migalastat-treated patients, resulting in frequencies of 17.2, 16.2 and 20.4 events /1.000 person years, respectively (Supplemental Table S5). Of note, in patients newly treated with agalsidase-alfa, all three events occurred in patients who had not previously been affected by any cerebrovascular event. In patients newly treated with agalsidase-beta or migalastat, one event occurred in previously unaffected patients (Supplemental Table S5). In long-term ERT-treated patients, only one event occurred in a patient treated with agalsidase-alfa (the patient already suffered from an event before baseline) resulting in a frequency of 5.4 events/ 1.000 person years (Supplemental Table S5).

Independently of the analyzed group, the frequencies of concomitant medications including RAAS blockers, diuretics and analgesics did not changed significantly over time (Supplemental Table S6). However, a nominal increase in the prescriptions of especially RAAS blockers was observed (Supplemental Table S6).

Since GI symptoms and FD-related pain belong to the most important symptoms affecting the quality of life in FD patients, we next examined the effects of the therapies on these relevant symptoms (Supplemental Table S7). Over time, we detected no significant changes for the frequencies of GI symptoms or FD-related pain. However, there was a nominal decrease of FD-related pain in newly agalsidase-alfa and migalastat-treated patients (Supplemental Table S7).

**Fig. 2:**
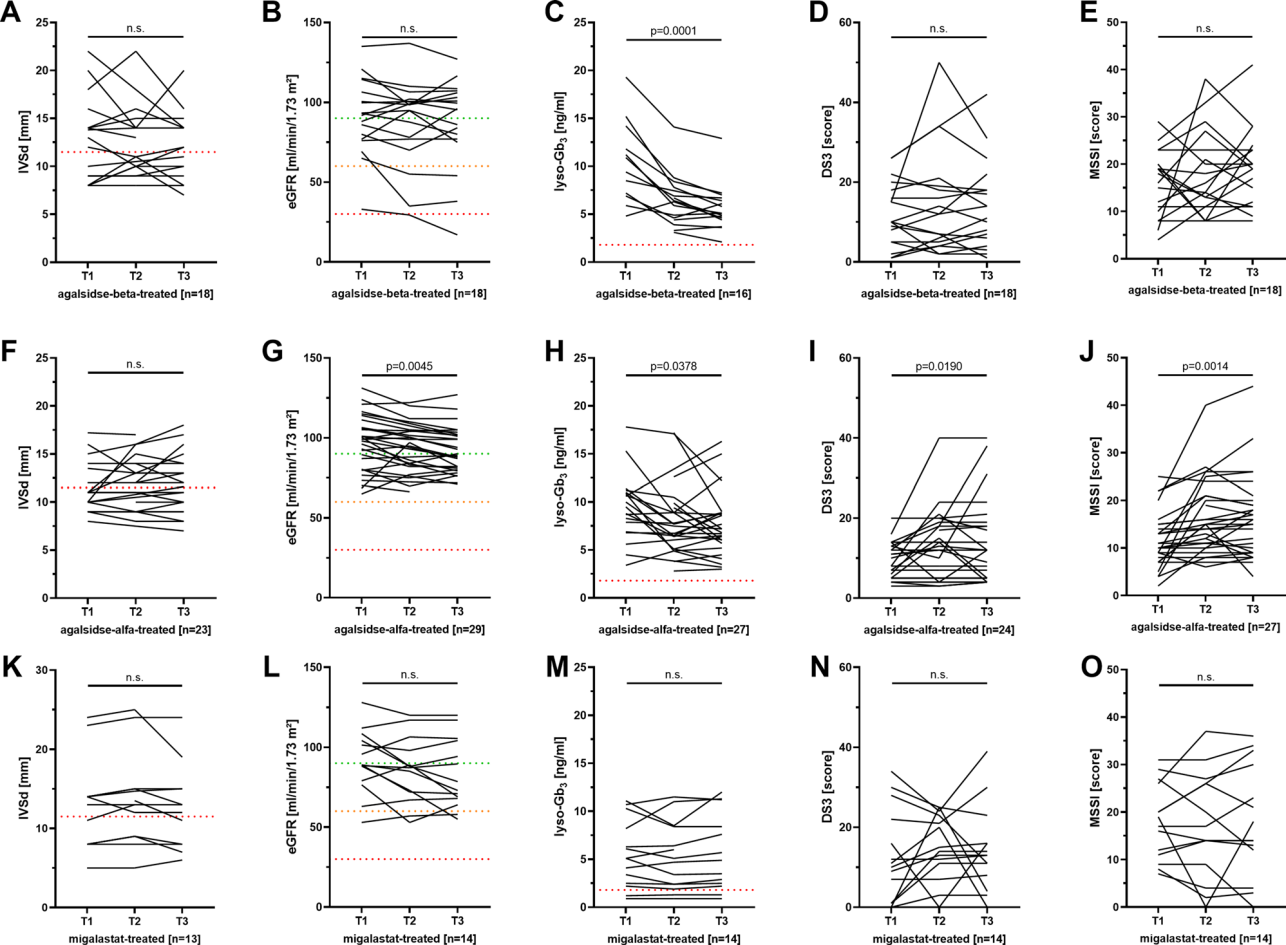
Individual disease course of newly treated patients. **A) - E)** Newly agalsidase-beta-treated patients. **F) - J)** Newly agalsidase-alfa-treated patients. **K) - O)** Newly migalastat-treated patients. DS3: Disease Severity Scoring System, eGFR: estimated glomerular filtration rate, IVSd: interventricular septum thickness in diastole, lyso-Gb_3_: globotriaosylsphingosine, MSSI: Mainz Severity Score Index, n.s.: not significant

**Fig. 3:**
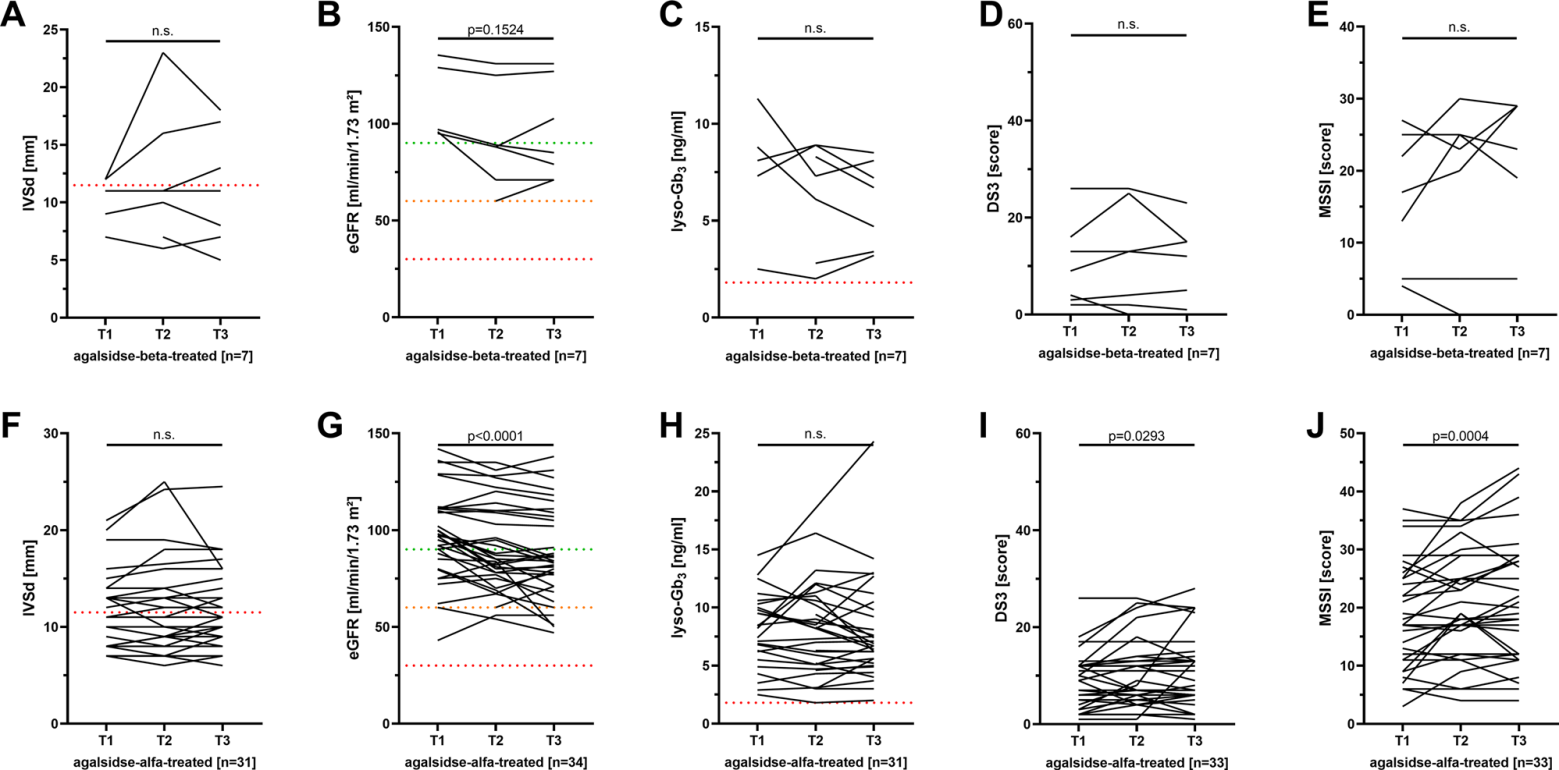
Individual disease course of long-term-treated patients. **A) - E)** Long-term agalsidase-beta-treated patients. **F) - J)** Long-term agalsidase-alfa-treated patients. DS3: Disease Severity Scoring System, eGFR: estimated glomerular filtration rate, IVSd: interventricular septum thickness in diastole, lyso-Gb_3_: globotriaosylsphingosine, MSSI: Mainz Severity Score Index, n.s.: not significant

During observation, no cardiac events including myocardial infarction, severe arrhythmia or heart failure and other FD-related cardiac events requiring hospitalization were reported. To assess the cardiac involvement over time, we analyzed IVSd as a marker for LVH (Figs. 2A, F and K and 3A and F). Overall, mean IVSd did not changed significantly between baseline and end of observation and, thus, was stable in all five groups (newly agalsidase-beta-treated group: -0.03 ± 0.64 mm/year; *p* = 0.8490; newly agalsidase-alfa-treated group: 0.00 ± 0.60 mm/year, *p* = 0.9867; newly migalastat-treated group: 0.02 ± 0.76 mm/year, *p* = 0.9108; long-term agalsidase-beta-treated group: -0.09 ± 1.32 mm/year; *p* = 0.8729, long-term agalsidase-alfa-treated group: 0.11 ± 0.47 mm/year; *p* = 0.2077). Independently of the treatment option, the presence of LVH at baseline had no effect on IVSd changes over time (all *p* < 0.05). eGFR was stable in newly agalsidase-beta- and migalastat treated patients (-0.6 ± 2.9 ml/min/1.73 m²/year, *p* = 0.2069 and − 1.6 ± 5.3 ml/min/1.73 m²/year, *p* = 0.2676, respectively) (Fig. 2B, L). Newly agalsidase-alfa-treated patients showed a slight but significant loss of eGFR by -1.5 ± 3.2 ml/min/1.73 m²/year (*p* = 0.0056; Fig. 2G). In addition, newly agalsidase-alfa treated patients also presented with a slight yearly ACR increase (10 ± 20 mg/g/year; *p* = 0.0296). eGFR in long-term agalsidase-beta-treated patients did not change significantly, but decreased also slightly in agalsidase-alfa-treated patients (-1.2 ± 5.3 ml/min/1.73 m²/year, *p* = 0.6875 and − 1.3 ± 3.9 ml/min/1.73 m²/year, *p* = 0.0187, respectively) (Fig. 3B, G).

To assess a change of the disease load, total DS3 and MSSI scores and also plasma lyso-Gb_3_ levels were analyzed. In newly agalsidase-beta and agalsidase-alfa-treated patients, plasma lyso-Gb_3_ levels decreased significantly (-1.7 ± 1.0 ng/ml/year, *p* = 0.0010 and − 0.5 ± 1.6 ng/ml/year, *p* = 0.0328, respectively; Fig. 2C, H). Plasma lyso-Gb_3_ levels in migalastat-treated patients as well as long-term agalsidase-beta and -alfa-treated patients remained stable (*p* = 0.3396, *p* = 0.4365 and *p* = 0.1056, respectively; Figs. 2M and 3C and H). DS3 and MSSI scores slightly increased in newly agalsidase-alfa- and long-term-agalsidase-alfa-treated patients over time (newly agalsidase-alfa-treated: 0.5 ± 1.6 scores/year; *p* = 0.0190 and 1.0 ± 1.1 scores/year; *p* = 0.0014; long-term agalsidase-alfa-treated: 0.6 ± 1.2 scores/year; *p* = 0.0293 and 1.2 ± 2.0 scores/year; *p* = 0.0004; Figs. 2I and J and 3I and J).

### Comparison between treatment regimes

**Fig. 4:**
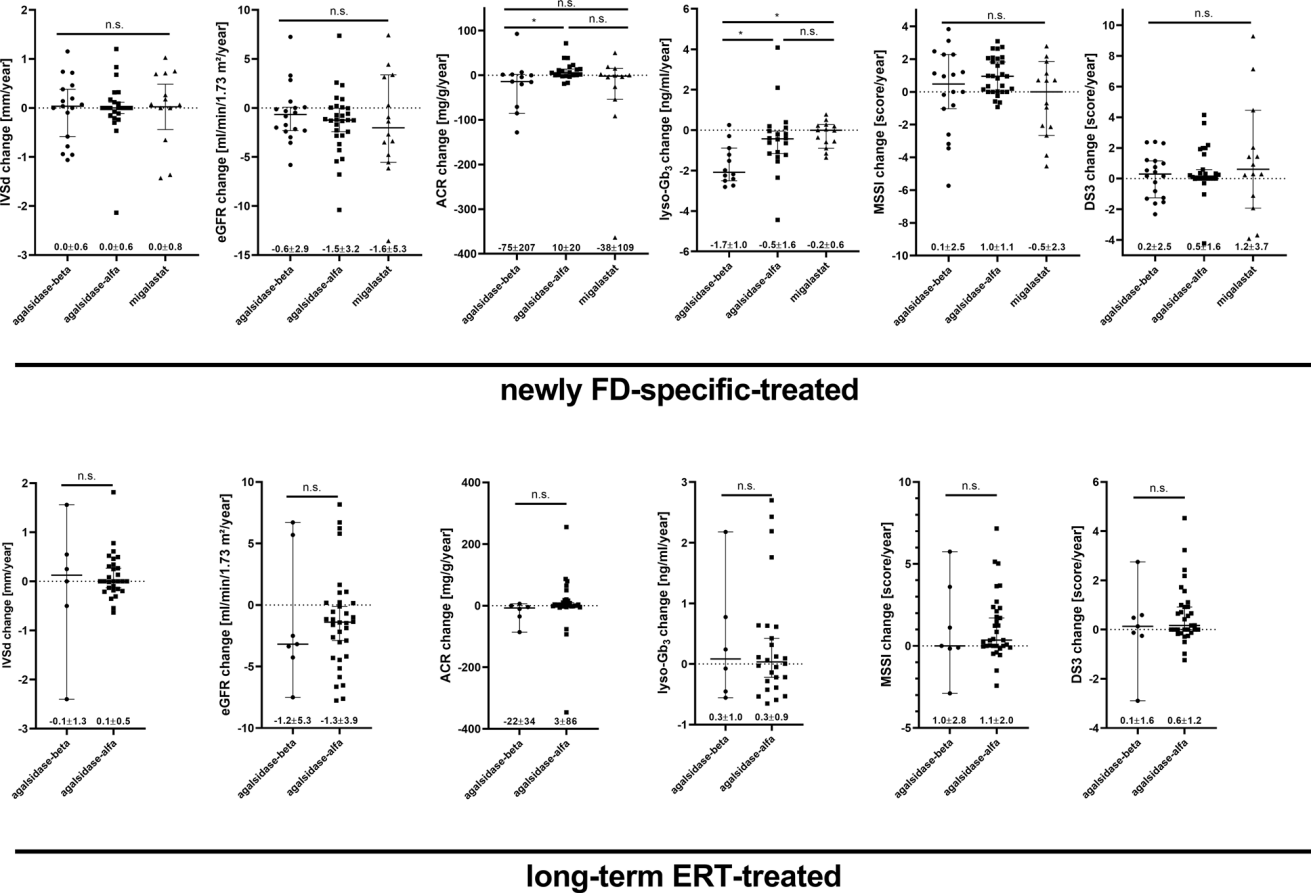
Overview of the yearly changes of IVSd, eGFR, ACR, lyso-Gb_3_, and total DS3 and MSSI under FD-specific therapy. ACR: albumin-creatinine-ratio, DS3: Disease Severity Scoring System, eGFR: estimated glomerular filtration rate, FD: Fabry disease, IVSd: interventricular septum thickness in diastole, lyso-Gb_3_: globotriaosylsphingosine, MSSI: Mainz Severity Score Index, n.s.: not significant. *p < 0.05

Finally, we compared efficacies of treatment options with each other (Fig. 4). Between the three groups of newly treated patients, we observed a significant difference for the reduction of plasma lyso-Gb_3_ (ANOVA: *p* = 0.0026, with agalsidase-beta versus migalastat: *p* = 0.0021, agalsidase-beta versus agalsidase-alfa: *p* = 0.0390, and agalsidase-alfa versus migalastat: *p* = 0.6559; Fig. 4). In addition, there was a slight but significant difference for changes in ACR over time between agalsidase-beta and agalsidase-alfa but not versus migalastat (ANOVA: *p* = 0.0192, with agalsidase-beta versus agalsidase-alfa: *p* = 0.0241, agalsidase-beta versus migalastat: *p* = 0.9999, agalsidase-alfa versus migalastat: *p* = 0.191; Fig. 4). For changes in IVSd, eGFR, DS3, and MSSI, we did not observe any differences between the treatment groups (neither between newly treated nor long-term-treated patients).

## Discussion

The aim of our multicenter study was to investigate the safety and efficacy of ERT and chaperone therapy on the disease progression in female FD patients and to compare the individual treatment regimens.

In short, our main findings are as follows: (1) treatment with agalsidase-beta, agalsidase-alfa and migalastat was safe, and severe adverse events were rare; (2) newly as well as long-term-treated patients presented a stable disease course over time; (3) no clinically relevant differences concerning treatment efficacy between agalasidase-beta, agalsidase-alfa and migalastat were detected.

### Safety of enzyme replacement and chaperone therapy

In terms of adverse events, the treatment with agalsidase-beta, agalsidase-alfa, and migalastat was safe. None of the treated patients suffered from severe adverse events under therapy. Only four (4.5%) patients under ERT required anti-allergic premedication before infusions, which could be tapered during ongoing treatment.

### Efficacy of enzyme replacement and chaperone therapy

#### Neurological symptoms and manifestations

The overall frequency of strokes/TIAs in untreated females is reported as ~ 16.7% [15]. To the best of our knowledge, no data concerning the frequencies of strokes/TIAs exist from clinical or observational studies with an approved dose of agalsidase-beta or agalsidase-alfa in females with FD. In our cohort, at baseline, the frequencies in ERT- and migalastat-treated patients (newly and long-term) were 10.3–23.5% and, thus, within the expected range. Independent of the treatment (ERT or chaperone), we observed no significant changes for cerebrovascular events over time in our cohort.

FD-related pain and GI symptoms are one of the most important manifestations in affected patients, significantly decreasing their quality of life. In long-term ERT-treated female patients, the presence of pain and GI symptoms remained stable over time. Consequently, pain medication in affected patients was also stable. Females newly treated with agalsidase-beta showed a trend for a reduction of GI symptoms, whereas those newly treated with agalsidase-alfa showed a trend for a reduction of FD-related pain. Females under migalastat showed a trend for a reduction of FD-related pain as well as GI symptoms. This is in accordance with current literature for agalsidase-beta [16], agalsidase-alfa [17, 18] and migalastat [19, 20].

#### Cardiac manifestations

Data from the Fabry Outcome Survey (FOS) registry demonstrated that, independently of sex, an early ERT initiation was associated with significant benefits in the reduction of cardiovascular events [21]. However, the beneficial effect of early initiation was more profound in patients ≤ 20 years of age at symptom onset [22]. These observations are in principle comparable to our findings, since independent of the chosen treatment (i.e. agalsidase-beta, agalsidase-alfa, or migalastat) no female suffered from cardiac events requiring hospitalization. Furthermore, all females presented with a stable septum thickness over time. Even those with LVH at T1 remained stable, indicating an achieved therapy goal [23, 24].

#### Renal manifestations

A progressive loss of eGFR is typical for patients with FD and increases morbidity and mortality. Affected untreated female patients may show an annual loss of renal function of up to 3 ml/min/1.73 m² [25]. The effects of FD-specific treatment on eGFR in females are heterogeneous and are mainly affected by mutation, age, CKD stage and comorbidities (such as hypertension) [2]. However, when analyzing the effects on renal function, the natural eGFR decline by ~ 1 ml/min/1.73 m²/year from the third decade of life [26] needs to be taken into account. Data from FOS demonstrated a stabilization of eGFR in females after 5 years of agalsidase-alfa treatment [25]. Comparable observations were made for females treated with agalsidase-beta [2, 27] and migalastat [28]. In our cohort, we detected a significant decline of eGFR over time in newly and long-term agalsidase-alfa-treated patients by 1.5 and 1.3 ml/min/1.73 m^2^/year, which is comparable to data from a previous multicenter study (classical woman: −1.4 ml/min/ 1.73 m² [29]). Since the observed reduction was within limits of natural eGFR decline typical for this age [26], kidney function in these patients can be interpreted as clinically stable. Under agalsidase-beta and migalastat there was a non-significant eGFR decline of -0.6 and − 1.6 ml/min/1.73 m²/year, respectively. This non-significant effect may be due to the lower number of patients (*n* = 7) and thus reduced statistical power.

### Additional aspects in the choice of therapy regimen

When initiating FD-specific therapy, several patient-specific aspects regarding the patient’s quality of life should be taken into account. The ability to integrate the therapy into the patient’s everyday life is particularly important. In the case of an amenable mutation, most females may prefer oral therapy with migalastat every other day. Adherence to therapy is a prerequisite in order to be able to follow the 2-hour abstinence from food before and after administration. If a non-amenable mutation is present, the duration of the infusion time (40 min [agalsidase-alfa] versus 1.5 h [agalsidase-beta] i.v.) is of primary importance in the choice of ERT - especially for the working patients.

Home infusions are possible with both ERTs, but at least in Germany present legal difficulties with regard to medical responsibility (delegation to nursing staff). Furthermore, in Germany economic aspects of therapy costs are playing an increasingly important role for the healthcare system. Doctors are called upon to decide on the most cost-effective therapy option, provided that the therapy efficiency of different therapy options is comparable. In Germany, the most cost-effective therapy option is currently chaperone therapy [€ 257,000 per year]. For ERT, agalsidase-beta [€339,000 per year for a patient with a mean body weight of 77.7 kg] is more cost-effective than agalsidase-alfa [€372,000 per year]. Additional studies are now warranted to assess the efficacy of the newly approved pegunigalsidase alfa (Chiesi, 309,000€ per year). However, although agalsidase-beta appears to be more cost effective, the shorter standard infusion time for agalsidase-alfa should not be ignored.

We conclude that treatment of females with agalsidase-beta, agalsidase-alfa or migalastat was safe. Independent of the chosen treatment regimen all patients presented with a stable disease course over time. In our cohorts, a comparison of therapy efficacies showed no relevant clinical differences between the analyzed groups.

## Limitations

Due to the COVID19 pandemic, the number of total recruited female patients with long-term follow-up data was limited. In addition, due to the retrospective approach, MRI data for cardiac and cerebral manifestations were also limited and, thus, not evaluated. Since only living patients were recruited at T3, no conclusions can be drawn concerning FD-related mortality. No data concerning enzymatic AGAL activities and quality of life were assessed in this study, which is a limitation. The group of long-term agalsidase-beta-treated patients is very small, and with regards to the restricted power, distinct conclusions on therapy efficacy should be drawn with caution. A comparison between patients receiving (any) ERT and migalastat should be interpreted carefully. Since due to the treatment modalities of migalastat (only patients with amenable missense mutation), patients receiving ERT might be more severe affected (patients with missense and nonsense mutations). Due to the small patient cohorts, some results might be influenced by individual outliers.

We did not find any indication that the newly-treated patients were generally less affected by FD. In accordance with the study protocol, patients with benign variants and variants of unclear significance were excluded. The cardiac late-onset mutation p.N215S was represented only once (migalastat group), excluding an effect of this mutation on outcomes. The slightly higher MSSI score in patients on migalastat could be due to a slightly higher age. However, these values should not be over interpreted, as the MSSI score was not established to document the disease burden in females, but only for males, and its use is therefore limited.

## Electronic supplementary material

Below is the link to the electronic supplementary material.

Supplementary Material 1

## Data Availability

The original contributions presented in the study are included in the article/Supplementary Material, further inquiries can be directed to the corresponding author.

## Abbreviations

AGAL/GLA: α-galactosidase A
ACR: Albumin-to-creatinine ratio
ANOVA: Analysis of variance
CKD-EPI: Chronic Kidney Disease-Epidemiology Collaboration equation
DS3: Disease Severity Scoring System
eGFR: Estimated glomerular filtration rate
ERT: Enzyme replacement therapy
FD: Fabry disease
Gb_3_: Globotriaoslyceramide
GI: Gastrointestinal
IVSd: Interventricular septum thickness in diastole
lyso-Gb_3_: Globotriaosylsphingosine
LVH: Left ventricular hypertrophy
MSSI: Mainz Severity Score Index
RAAS: Renin-angiotensin-aldosterone-system
RR: Relative risk
TIA: Transient ischemic attack
