# Supplementary material for: Impact of enzyme replacement therapy and migalastat on disease progression in females with fabry disease
Source: Orphanet J Rare Dis. 2025 Feb 20;20:79. doi: 10.1186/s13023-025-03600-y (PMC11843741; doi:10.1186/s13023-025-03600-y)
Supplement: Supplementary file 1 — Supplementary Material 1 [file 13023_2025_3600_MOESM1_ESM.docx]

| **Supplemental Table 1: Overview of identified α-galactosidase A mutations within the recruited patients.** | | |
| --- | --- | --- |
| **mutations** | **missense** | **nonsense/insertions/deletions/intronic** |
| ***newly treated*** | | |
| **agalsidase-beta** | p.C63Y, p.A160P, p.I317T, p.I319T, p.G328R, p.R342Q, p.L344P, p.S345P | p.Y173X, p.Y216X, p.R220X, p.T340X, c.723dupT, c.744_745del, Deletion Exon 5-7 |
| **agalsidase-alfa** | p.M42V, p.L45P, p.G138R, p.C202Y, p.S247P, p.G328R, p.G361R | p.Y151X, p.Y173X, p.W204X, p.Q280X, p.T287X, p.R310X, p.R220X, p.R342X, p.W349X, p.Q357X, IVS2 +1G>T, c.369+1G>T, c.714ins7bp, c.1167 dup T, c.1168ins T, Deletion Exon 5-7 |
| **migalastat** | p.M42T, p.R118C, p.T194I, p.K213M, p.N215S, p.I219M, p.P259R, p.G271D, p.T282I, p.L294S, p.N320I | none |
| ***long-term ERT-treated*** | | |
| **agalsidase-beta** | p.D266Y, p.S345P | p.W262X, c.762ins282bp, c.1232delG, IVS2+1G>A |
| **agalsidase-alfa** | p.M42T, p.E66K, p.G138R, p.T194I, p.S247P, p.P265S, p.N278K, p.A288D, p.R301Q, p.S345P, p.A350P, p.L388P | p.R220X, p.R227X, p.R301X, p.W340X, p.R342X, c.364delA, c.370-2A>G,  c.718_719delAA, c.744-745 delTA, c.762ins282bp, c.1167dupT, c.1168insT |
| ERT: enzyme replacement therapy. | | |

| **Supplemental Table 2: Clinical work-up performed at each visit.** | |
| --- | --- |
| **organ/domain** | **investigation/ measure** |
| **Medical history (mandatory)** | date of birth, date of medical check-up, time of ERT start, enzyme compound, dose and frequency of ERT, premedication, symptoms (diarrhea, abdominal pain, hypohidrosis, cornea verticillata, tinnitus, hearing loss, fatigue, FD-related pain, TIA, stroke, dyspnea, NYHA class, myocardial infarction, severe arrhythmia, pacemaker implantation, dialysis/kidney transplantation), onset of symptoms, family history, dose of current concomitant medication |
| **Physical examination (mandatory)** | routine examination, height, weight, BP, heart rate, angiokeratoma |
| **Cardiology** |  |
| mandatory | electrocardiography, echocardiography (LVEDD, LVESD, LVSd, LVPWd, EF, diastolic function) |
| optional | cardiac MRI (standard, late enhancement imaging) |
| **Neurology** |  |
| mandatory | examination, history, polyneuropathy tests |
| optional | cerebral MRI |
| **Nephrology** |  |
| mandatory | albuminuria (albumin-to-creatinine ratio), serum creatinine, eGFR (CKD-EPI), urine analysis |
| optional | cystatin C-based GFR |
| **Laboratory** |  |
| mandatory | α-galactosidase A mutation, lyso-Gb_3_, standard laboratory setting |
| optional | α-galactosidase A activity |
| **Disease severity scores (mandatory)** | Disease Severity Scoring System (DS3), Mainz severity score index (MSSI) |
| TIA, transient ischemic attack; NYHA, New York Heart Association; LVEDD, left ventricular end-diastolic diameter; LVESD, left ventricular end-systolic diameter; LVSd, left ventricular septum in diastole; LVPWd, left ventricular posterior wall in diastole; EF, ejection fraction; MRI, magnetic resonance imaging; CKD-EPI, CKD-Epidemiology Collaboration. | |

| **Supplemental Table 3: Quality control of assessed data for analyzed patients (n=102).** | | |
| --- | --- | --- |
| **organ/domain** | **investigation/ measure, n (%)** | **combined work-up (%)** |
| **medical history** | age: 102 (100.0); BMI: 101 (99.0); SBP: 97 (95.1); DBP: 97 (95.1); treatment status: 102 (100.0) | 97.8 |
| **laboratory parameters** | genotype: 102 (100.0); plasma lyso-Gb_3_: 75 (73.5) | 86.7 |
| **concomitant medication** | RAAS blockers: 97 (95.1); diuretics: 98 (96.1); analgesics: 93 (91.2) | 94.1 |
| **clinical presentation** | angiokeratoma: 101 (99.0); edema: 101 (99.0); gastrointestinal symptoms: 94 (92.1); FD-related pain: 102 (100.0); fatigue: 71 (69.6); ever stroke/TIA: 102 (100.0); MSSI: 99 (97.0); DS3: 95 (93.1) | 93.7 |
| **cardiac measures** | IVSd: 87 (85.3); pacemaker: 102 (100.0); ICD: 102 (100.0), myocardial infarction: 102 (100.0) | 96.3 |
| **renal measures** | ACR: 84 (82.3); serum creatinine: 99 (97.0); eGFR_creat_: 99 (97.0); dialysis: 102 (100.0); kidney transplantation: 102 (100.0) | 95.3 |
| **overall data completeness** |  | **94.0** |
| ACR: albumin/creatinine-ratio; DBP: diastolic blood pressure; DS3: Disease Severity Scoring System; eGFR: estimated glomerular filtration rate; FD: Fabry disease; ICD: implantable cardioverter device; IVSd: interventricular septum thickness in diastole; lyso-Gb_3_: globotriaosylsphingosine, MSSI: Mainz Severity Score Index; RAAS: renin-angiotensin-aldosterone-system; SBP: systolic blood pressure; TIA: transitory ischemic attack. | | |

| **Supplemental Table 4: Overview of the median intervals between visits.** | | | |
| --- | --- | --- | --- |
| **group** | **T1 to T2 (months)** | **T2 to T3**  **(months)** | **Overall (T1 to T3)**  **(months)** |
| ***newly treated*** | | | |
| **agalsidase-alfa** | 38 [10 to 132] | 25 [8 to 82] | 76 [15 to 167] |
| **agalsidase-beta** | 26 [6 to 146] | 23 [12 to 128] | 61 [24 to 170] |
| **migalastat** | 19 [6 to 57] | 16 [6 to 33] | 36 [21 to 73] |
| ***long-term treated*** | | | |
| **agalsidase-alfa** | 25 [11 to 165] | 22 [9 to 45] | 47 [22 to 183] |
| **agalsidase-beta** | 25 [12 to 97] | 25 [9 to 45] | 50 [23 to 142] |
| Values are given as median with min to max. | | | |

| **Supplemental Table 5: Frequencies of transient ischemic attacks and strokes during follow-up.** | | | | | |
| --- | --- | --- | --- | --- | --- |
|  | **patients with events before T1, n (%)** | **events between T1 and T3, n (%)** | **patients with de novo events, n (%)** | **total observation time between T1 and T3 (years)** | **frequency**  **(events/ 1.000 patient years)** |
| ***newly treated*** | | | | | |
| **agalsidase-beta [n=18]** | 3 (16.7) | 2 (11.1) | 1 (5.5) | 116 | 17.2 |
| **agalsidase-alfa [n=29]** | 3 (10.3) | 3 (10.3) | 3 (10.3) | 185 | 16.2 |
| **migalastat [n=14]** | 3 (21.4) | 1 (7.1) | 1 (7.1) | 49 | 20.4 |
| ***long-term ERT-treated*** | | | | | |
| **agalsidase-beta [n=7]** | 1 (14.3) | 0 (0.0) | 0 (0.0) | 39 | n.d. |
| **agalsidase-alfa [n=34]** | 8 (23.5) | 1 (2.9) | 0 (0.0) | 186 | 5.4 |
| Patients with de novo events are defined as patients previously unaffected by any cerebrovascular event. ERT: enzyme replacement therapy. | | | | | |

| **Supplemental Table 6: Changes in concomitant medication during follow-up.** | | | | |
| --- | --- | --- | --- | --- |
|  | **treated at baseline (T1), n (%)** | **changes, n** | **treated at end of observation (T3), n (%)** | **relative risk [95% CI]** |
| ***newly treated*** | | | | |
| **RAAS blocker** | | | | |
| *agalsidase-beta* | 3 (18.7) | +5/-0 | 8 (50.0) | 2.27 [0.96 to 6.61] |
| *agalsidase-alfa* | 13 (48.1) | +7/-2 | 18 (64.3) | 1.45 [0.85 to 2.49] |
| *migalastat* | 6 (42.8) | +1/-0 | 7 (50.0) | 1.16 [0.55 to 2.55] |
| **diuretics** | | | | |
| *agalsidase-beta* | 1 (6.2) | +3/-0 | 4 (25.0) | 2.78 [0.80 to 15.65] |
| *agalsidase-alfa* | 4 (14.3) | +2/-4 | 2 (7.1) | 0.72 [0.44 to 1.66] |
| *migalastat* | 2 (14.3) | +3/-1 | 4 (28.6) | 1.64 [0.66 to 5.87] |
| **analgesics** | | | | |
| *agalsidase-beta* | 3 (20.0) | +4/-1 | 6 (37.5) | 1.70 [0.75 to 4.91] |
| *agalsidase-alfa* | 1 (3.8) | +2/-0 | 3 (11.5) | 2.08 [0.69 to 11.58] |
| *migalastat* | 2 (14.3) | +0/-1 | 1 (7.1) | 0.72 [0.37 to 2.43] |
| ***long-term ERT-treated*** | | | | |
| **RAAS blocker** | | | | |
| *agalsidase-beta* | 3 (50.0) | +1/-0 | 4 (66.7) | 1.40 [0.44 to 4.34] |
| *agalsidase-alfa* | 14 (41.2) | +4/-1 | 17 (50.0) | 1.20 [0.74 to 1.99] |
| **diuretics** | | | | |
| *agalsidase-beta* | 1 (16.7) | +2/-0 | 3 (50.0) | 2.50 [0.65 to 14.38] |
| *agalsidase-alfa* | 5 (14.7) | +4/-1 | 8 (23.5) | 1.37 [0.75 to 3.07] |
| **analgesics** | | | | |
| *agalsidase-beta* | 1 (16.7) | +1/-0 | 2 (33.3) | 1.67 [0.48 to 9.57] |
| *agalsidase-alfa* | 5 (15.6) | +1/-1 | 5 (15.6) | 1.00 [0.58 to 2.19] |
| ERT: enzyme replacement therapy (includes treatment with agalsidase-alfa or -beta), FD: Fabry disease, RAAS: renin-angiotensin-aldosterone system. | | | | |

| **Supplemental Table 7: Changes in gastrointestinal symptoms and FD-related pain under FD-specific therapy.** | | | | |
| --- | --- | --- | --- | --- |
|  | **at baseline (T1), n (%)** | **changes, n** | **at end of observation (T3), n (%)** | **relative risk [95% CI]** |
| ***newly treated*** | | | | |
| **GI symptoms** | | | | |
| *agalsidase-beta* | 6 (40.0) | +1/-3 | 4 (26.7) | 0.75 [0.38 to 1.62] |
| *agalsidase-alfa* | 5 (18.5) | +3/-2 | 6 (22.2) | 1.13 [0.62 to 2.51] |
| *migalastat* | 5 (45.4) | +0/-2 | 3 (27.3) | 0.68 [0.30 to 1.64] |
| **FD-related pain** | | | | |
| *agalsidase-beta* | 12 (66.7) | +1/-1 | 12 (66.7) | 1.00 [0.47 to 1.90] |
| *agalsidase-alfa* | 18 (62.1) | +0/-7 | 11 (37.9) | 0.66 [0.41 to 1.06] |
| *migalastat* | 9 (64.3) | +0/-4 | 5 (35.7) | 0.55 [0.24 to 1.18] |
| ***long-term ERT-treated*** | | | | |
| **GI symptoms** | | | | |
| *agalsidase-beta* | 0 (0.0) | +1/-0 | 1 (14.3) | n.a. |
| *agalsidase-alfa* | 4 (11.8) | +2/-2 | 4 (11.8) | 1.00 [0.57 to 2.39] |
| **FD-related pain** | | | | |
| *agalsidase-beta* | 4 (57.1) | +0/-1 | 3 (42.8) | 0.75 [0.24 to 2.15] |
| *agalsidase-alfa* | 25 (73.5) | +2/-3 | 24 (70.6) | 0.93 [0.51 to 1.52] |
| ERT: enzyme replacement therapy, FD: Fabry disease, GI: gastrointestinal symptoms (including the presence of diarrhea and/or abdominal pain), n.a.: not applicable. | | | | |
